# Transcriptome of *Nosema ceranae* and Upregulated Microsporidia Genes during Its Infection of Western Honey Bee (*Apis mellifera*)

**DOI:** 10.3390/insects13080716

**Published:** 2022-08-09

**Authors:** Yi-Hsuan Li, Zih-Ting Chang, Ming-Ren Yen, Yu-Feng Huang, Tzu-Han Chen, Ju-Chun Chang, Ming-Cheng Wu, Yu-Liang Yang, Yue-Wen Chen, Yu-Shin Nai

**Affiliations:** 1 Department of Entomology, National Chung Hsing University, Taichung City 40227, Taiwan; 2 Department of Biotechnology and Animal Science, National Ilan University, Yi-Lan City 26047, Taiwan; 3 Department of Computer Science and Engineering, Yuan-Ze University, Tao-Yuan City 32003, Taiwan; 4 Agricultural Biotechnology Research Center, Academia Sinica, Taipei City 11529, Taiwan; 5 Biotechnology Center in Southern Taiwan, Academia Sinica, Tainan 711010, Taiwan

**Keywords:** common DEGs, stage-specific genes, transcriptome, *Nosema ceranae*, *Apis mellifera*

## Abstract

**Simple Summary:**

In this study, the gene expression profile of a honey bee microsporidium, *Nosema* *ceranae*, was investigated at 5, 10, and 20 days post-infection. Based on transcriptome data, the common DEGs and stage-specific genes were identified and validated. Our data reveal that the gene expression of *N. ceranae* during infection is highly related to the mechanisms of gene transcription, protein synthesis, and structural proteins. This information could be a reference for the control of nosemosis at the genetic level in apiculture.

**Abstract:**

*Nosema ceranae* is one of the fungal parasites of *Apis mellifera*. It causes physical and behavioral effects in honey bees. However, only a few studies have reported on gene expression profiling during *A. mellifera* infection. In this study, the transcriptome profile of mature spores at each time point of infection (5, 10, and 20 days post-infection, d.p.i.) were investigated. Based on the transcriptome and expression profile analysis, a total of 878, 952, and 981 differentially expressed genes (DEGs) (fold change ≥ 2 or ≤ −2) were identified in *N. ceranae* spores (NcSp) at 5 d.p.i., 10 d.p.i., and 20 d.p.i., respectively. Moreover, 70 upregulated genes and 340 downregulated genes among common DEGs (so-called common DEGs) and 166 stage-specific genes at each stage of infection were identified. The Gene Ontology (GO) analysis indicated that the DEGs and corresponding common DEGs are involved in the functions of cytosol (GO:0005829), cytoplasm (GO:0005737), and ATP binding (GO:0005524). Furthermore, the pathway analysis found that the DEGs and common DEGs are involved in metabolism, environmental information processing, and organismal systems. Four upregulated common DEGs with higher fold-change values, highly associated with spore proteins and transcription factors, were selected for validation. In addition, the stage-specific genes are highly involved in the mechanism of pre-mRNA splicing according to GO enrichment analysis; thus, three of them showed high expression at each d.p.i. and were also subjected to validation. The relative gene expression levels showed a similar tendency as the transcriptome predictions at different d.p.i., revealing that the gene expression of *N. ceranae* during infection may be related to the mechanism of gene transcription, protein synthesis, and structural proteins. Our data suggest that the gene expression profiling of *N. ceranae* at the transcriptomic level could be a reference for the monitoring of nosemosis at the genetic level.

## 1. Introduction

Microsporidia are considered primitive eukaryotic and obligate intracellular parasites; they have 70 S ribosomes of small subunit (SSU) and large subunit (LSU) rRNA, which is similar to eukaryotes, but they lack mitochondria, peroxisomes, and centrioles. Nonetheless, molecular phylogeny shows that microsporidia are regarded as highly specialized parasitic fungi [1,2,3,4,5,6]. Nosemosis is a serious and common microsporidial disease of adult honey bees that is mainly caused by two species of microsporidians, *Nosema apis* and *Nosema ceranae* [7,8]. *N. ceranae* and *N. apis* are described as midgut pathogens found in adult honey bees that reproduce only in midgut epithelial cells [9]. There are no clear outward symptoms of nosemosis; however, whitish and swollen midguts are observed in heavily infected honey bees, honey bees become weak, and colonies can even collapse.

In general, *Nosema* spp. have two distinct phases in their life cycle, including the proliferative phase (merogony) and sporogonic phase (sporogony). The morphology and life cycle of *N. apis* and *N. ceranae* are quite similar, and previous studies have also proven that *N. apis* and *N. ceranae* can infect *Apis mellifera* [9,10,11]. However, some different features are found between *N. apis* and *N. ceranae*, including genome size, temperature resilience, survival, and impacts on infected honey bees [9,12,13,14]. Compared with *N. apis*, *N. ceranae* has a higher virulence and appears to successfully compete with *N. apis* in many *A. mellifera* populations globally. Therefore, *N. ceranae* manifests as a long-term chronic infection in warmer areas and then causes colony loss [15,16,17].

As mentioned above, *N. ceranae* mainly infects the midgut tissues of honey bees, and many studies have demonstrated the impacts of an *N. ceranae* infection on *A. mellifera*. Once honey bees are infected with *N. ceranae*, immunity and physiological conditions decrease, eventually leading to colony weakness and losses [18,19]. Because of the pressure of the lack of nutrition and energy, a higher hunger level is observed in infected honey bees, and more sugar is consumed [19,20,21]. Moreover, *N. ceranae* replicates with considerable energy taken from the host, causing malnutrition and increased mortality in honey bees [22]. The expression profile of the genes associated with the carbohydrate metabolism of *A. mellifera* are changed during the process of an *N. ceranae* infection. Besides, severe physiological effects caused by an infection with *N. ceranae*, including the expression of age-inappropriate behaviors, a reduced flying capability, and a shortened lifespan, were also reported [23]. It was reported that an infection with *N. ceranae* does not cause large compositional changes to the honey bee midgut microbiota, but the relative abundances of Proteobacteria and Firmicutes were significantly changed under a severe *N. ceranae* infection, indicating the dysbiosis state of the bacteriome in the honey bee gut [24]. In addition, it has also been reported that co-exposure to pesticides, such as fipronil or thiamethoxam, and *N. ceranae* decreased the survival rate of honey bees [25]. In terms of microbiota composition, co-exposure to pesticides and *N. ceranae* also negatively influenced the abundance of Alphaproteobacteria, but increased the Gammaproteobacteria abundance, indicating that the pesticides and *N. ceranae* are associated with the pathobiome status of honey bees [25]. Even though some bacterial species exhibited inhibitory activity on *N. ceranae,* it has been reported that the enhancement of *Bifidobacterium* spp. in the hindgut after *N. ceranae* infection may not inhibit nosemosis [25,26,27].

Previous studies have examined the gene expression profile and corresponding differentially expressed genes of *N. ceranae*-infected *A. mellifera* [28,29,30]. However, few studies have emphasized either the comprehensive gene expression of *N. ceranae* during its infection of *A. mellifera* or the common DEGs, which are important to *N. ceranae* for completing its life cycle [31]. Moreover, the genome of *N. ceranae* has been studied and reported for a better understanding of the architecture, regulation, and evolution of this pathogen [31,32]. From our previous study, the identification of *N. ceranae*-specific genes during its infection of *A. mellifera* by suppressive subtractive hybridization (SSH) has been performed [33], but comprehensive data on the gene expression profile during the infection process are still unavailable. In this study, we attempted to reveal the transcriptomic profiling of *N. ceranae* during its infection of *A. mellifera* to complete the information on the infection cycle of this parasite. To this end, a transcriptomic approach at different *N. ceranae* infection stages in *A. mellifera* was followed, and common DEGs and stage-specific genes among different infectious periods were identified and validated. This study provides information to better understand the mechanism of *N. ceranae* and guide basic disease-control studies.

## 2. Materials and Methods

– Honey bees

Worker honey bees were collected from *A. mellifera* colonies in apiaries at the National Chung Hsing University (NCHU, Taiching, Taiwan). Each honey bee colony had six to eight frames and the presence of a normal spawning queen. Brood cells of honeycombs from the six colonies, which were capped at more than 70–80%, were selected and incubated at 34 °C until honey bees emerged. The newly emerged adult honey bees were collected in a rearing cage and supplied with 50% syrup for the following experiments.

– Artificial infection of Nosema ceranae

Mature *N. ceranae* spores were collected from the midgut tissues of honey bees from apiaries at the NCHU (National Chung Hsing University, Taichung, Taiwan) and purified by the protocol proposed by Huang et al., 2007 [34]. Briefly, the midgut tissues of honey bees were collected in 500 μL 1 × TE buffer (10 mM Tris, 1 mM EDTA, pH 7.5) (Bioman, New Taipei, Taiwan) and homogenized with a pestle. The homogenized tissues were then filtered with 100 mesh stainless steel wire cloth. The filtrate was centrifuged at 1000× *g* for 10 min, and the supernatant and tissue debris were removed. Then, the pellet was resuspended in 500 μL 1 × TE buffer. The wash step was repeated to remove any remaining tissue debris. The spore suspension was centrifuged at 16,000× *g* for 3 min at 4 °C, and the collected spores were counted with a haemocytometer (Paul Marienfeld GmbH & Co. KG, Baden-Württemberg, Germany). The concentration of the mature spore suspension was adjusted to 1 × 10^5^ spore/2 μL for the infection of honey bees [35].

In the *N.* *ceranae*-infected group, the newly emerged honey bees were fed 2 μL of 50% sugar syrup with *N. ceranae* spores (1 × 10^5^ spores per honey bee), while the honey bees in the control group were fed 2 μL of 50% sugar syrup. Each group contained fifty honey bees in one rearing cage, which were supplied with 50% syrup and incubated at 34 °C until sampling. Samples for both RNA-seq and the detection of *N. ceranae* infections were selected in triplicate, and qPCR validation for gene expression was repeated in four replicates. The survival rates of the infected and control groups were observed and recorded daily. A Kaplan–Meier survival curve analysis was performed using SPSS (Norman H. Nie, C. Hadlai (Tex) Hull and Dale H. Bent, IBM SPSS Statistics version 20, Palo Alto, CA, USA).

– Confirmation of Nosema ceranae infection

Before the experiment, multiplex PCR [36] was performed to confirm the absence of *Nosema* spp. infection in newly emerged adult honey bees (Supplementary Figure S1). To confirm the *N. ceranae* infection, three midgut tissues of *N. ceranae*-infected bees and noninfected honey bees were collected in 1 × TE buffer. The midgut tissues were homogenized with a pestle, and the tissue suspension was subjected to observation, spore counting, and DNA extraction. Mature spores were observed under 400× light microscopy (WHITED, Taiwan) and counted by a haemocytometer (Paul Marienfeld GmbH & Co. KG, Baden-Württemberg, Germany). A total of 100 μL of homogenized tissue suspension was subjected to DNA extraction by the EDNA HISPEX (Easy DNA High-Speed Extraction) tissue kit (Fisher Biotec, Stirling, Australia) according to the user manual. The DNA sample was then subjected to PCR detection with the microsporidia universal primer set 18f/1537r, and the Hb18f/Hb18r primer set was used as an internal control (Supplementary Table S1). For the PCR, each 20 μL PCR contained 10 μL 2× SuperRed PCR Master mix (BIOTOOLs, New Taipei, Taiwan), 1 μL 100 mM of each primer, and 1 μL template DNA. PCR amplification was performed by a MiniAmp™ Thermal Cycler (Thermo Scientific, Waltham, MA, USA) with the following program: the thermal cycle was preheated to 95 °C for 10 min, followed by 35 cycles of 95 °C for 15 s, 55 °C for 30 s, and 72 °C for 1 min 30 s, and then a final extension was performed of 72 °C for 10 min and storage at 20 °C. The PCR product was analyzed by electrophoresis on a 1% agarose gel in 1 × Tris-acetate-EDTA (TAE) buffer.

– Preparation of RNA samples for next-generation sequencing

Based on the data of the survival curve and mature spore counts, midgut tissues from three honey bees were collected at 0 (3 h), 5, 10, and 20 days post-infection (d.p.i.) and purified *N. ceranae* mature spores (1 × 10^8^) were homogenized by a Tissue Lyser II (QIAGEN, Solingen, Germany) at f = 30/s for 1 min. This procedure was repeated at three time points. The total RNA was extracted using TRIzol reagent (Invitrogen Life Technologies, Waltham, MA, USA) following the user’s manual. The extracted RNA sample was then purified using a Locking Gel (BIOTOOLS, New Taipei, Taiwan). The RNA purity and integrity were checked by a Nanodrop 1000 Spectrophotometer V3.5 (Thermo Scientific, Waltham, MA, USA) and Qubit 2.0 Fluorometer (Invitrogen Life Technologies, Waltham, MA, USA), respectively. Total RNA samples of three biological repeats were pooled and used for library construction. The size distribution of each RNA sample was determined by Fragment Analyzer Systems (Agilent, Santa Clara, CA, USA). The RNA sample was then used to construct a library for transcriptome sequencing.

– RNA-Seq library construction and sequencing

The mRNA from each sample was further purified for sequencing library construction using an Ultra RNA Library Prep Kit (NEB, Essex, MA, USA) according to the manufacturer’s protocol. Briefly, poly-A-containing mRNA was purified by oligo d (T) magnetic beads. During the final step of mRNA purification, RNA was fragmented and primed with NEBNext random primers for cDNA synthesis. After mRNA was reverse-transcribed, end-repair and adaptor ligation (NEBNext Adaptor) were performed. Finally, DNA fragments were selectively enriched by PCR and then qualified and quantified by Fragment Analyser Systems (Agilent, Santa Clara, CA, USA) and qPCR, respectively, for the DNA library quality control analysis. The libraries were then pooled and sequenced by the Illumina MiSeq sequencer with a paired-end (PE) technology of 301 bp in length to generate high-throughput transcriptome sequencing data. Sequencing adaptors were trimmed using Trimmomatic [37] from raw PE reads, and then a read quality check was performed by PRINseq [38] from trimmed PE reads. To confirm the pathogen load in the sequencing data of the newly emerged adult honey bees, the trimmed PE reads were mapped to the genomes of *Nosema apis* and 6 other viruses.

– Differentially expressed gene (DEG) analysis

The raw PE reads were trimmed by Trimmomatic [37], and then the clean reads were obtained. The quality reads were mapped to the *N. ceranae* genome index (ASM98816v1) using HISAT2 with default parameters, and the corresponding gene annotation information (GCF_000988165.1_ASM98816v1_genomic.gff) from the National Biotechnology Information Center (NCBI) (https://www.ncbi.nlm.nih.gov/ (accessed on 19 January 2021)) was used to obtain the *N. ceranae*-related PE reads for further analysis. Differentially expressed genes (DEGs) among experimental groups were analyzed via Cuffdiff, and the gene expression heatmap was generated by the R package [39] (Supplementary Figure S2). For the DEG analysis, genes with a zero FPKM value from each group of the analysis were filtered out. To facilitate logarithmic transformation, a value of 1 was added to the FPKM value of the remaining genes. Raw signals (FPKM t 1) were then transformed using log2-based transformation. A statistical test of fold changes was performed, and a significant difference in the expression of these two groups was defined as a log2 ratio ≥ 1 or ≤ −1 (fold change ≥ 2 or ≤ −2). The DEGs from each comparison (NcSp/5 d.p.i., NcSp/10 d.p.i., and NcSp/20 d.p.i.) were further compared by Venny 2.1.0 (Juan Carlos Oliveros, Madrid, Spain) [40] to obtain the common DEGs of *N. ceranae* in different infection stages. The DEGs and common DEGs were further used in the GO enrichment analysis and submitted to the KEGG Automatic Annotation Server (KAAS, http://www.genome.jp/tools/kaas/ (accessed on 23 April 2021)) for gene orthologue assignment and pathway mapping [41]. The selected *N. ceranae* common DEGs were further analyzed by EMB BLAST (European Molecular Biology Network—Swiss node) for a functional homology search. The results with an E-value < 10^−4^ were recorded for further discussion of functional proteins.

– qRT–PCR validation

For validation, three midgut tissues of infected honey bees were collected into 300 μL of TE buffer at 0.5 (12 h), 1, 3, 5, 10, 15, and 20 d.p.i., with four replicates, and were homogenized for RNA extraction. Four replicates of 1 × 10^8^ purified mature *N. ceranae* spores were also subjected to RNA extraction. The total RNA for qRT–PCR validation was extracted from 200 μL of homogenized midgut tissue and the *N. ceranae* mature spore sample using the GENEzol™ TriRNA Pure Kit (Geneaid, New Taipei, Taiwan) as described in the user manual. The concentration and quantity of the total RNA were measured using a Nanodrop™ spectrophotometer (Thermo Scientific, Waltham, USA). Each RNA sample was adjusted to 1 µg/mL for cDNA synthesis by BioDiamond RT–PCR mix (Origin Pure BioSci & Tech.Co., Yilan, Taiwan) as described in the user manual. Four highly upregulated common DEGs, AAJ76_1600052943, AAJ76_2000149161, AAJ76_2000141845, and AAJ76_500091146, and three highly expressed stage-specific genes, AAJ76_5000026485, AAJ76_2400020190, and AAJ76_2300035467, were selected for RT–qPCR validation. β-Tubulin from *N. ceranae* served as the internal control. Real-time PCR was performed by iQ™ SYBR^®^ Green Supermix (Bio–Rad, Hercules, CA, USA) in a CFX Connect Real-Time PCR Detection System (Bio–Rad, Hercules, CA, USA). qRT–PCR was performed as follows: 95 °C for 3 min, followed by 40 cycles of 95 °C for 10 s, 60 °C for 30 s, and 72 °C for 45 s. The relative gene expression levels for common DEGs were calculated by the 2-△△Ct method, and stage-specific genes were calculated by the 2-△Ct method [42].

– Statistical analysis

Statistical analysis and plotting were performed with R 4.0.3 [43] and the heatmap, ggplot2, and ggpubr [39,44,45] packages. The statistical analysis of the Kaplan–Meier survival curves was performed using the R packages survminer [46] and survival [47]. The statistical analysis of the spore count and qRT–PCR data was performed using one-way ANOVA with SPSS^®^ [48].

## 3. Results

### 3.1. N. Ceranae Infection

**Figure 1:**
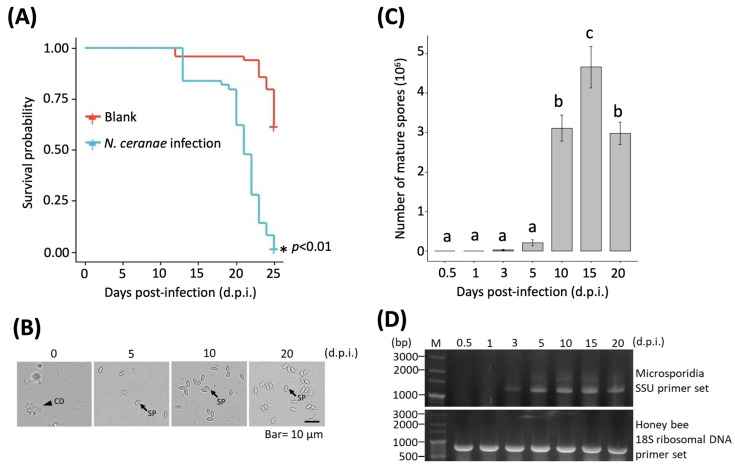
Observation and molecular identification of *N. ceranae* infection. (**A**) Kaplan–Meier survival curve (* = significant difference); (**B**) observation of mature spores; (**C**) mature spore counts (Different letters indicate significant differences (*p* < 0.05)); and (**D**) molecular detection of *N. ceranae* infection at 0.5 (12 h), 1, 3, 5, 10, 15, and 20 d.p.i.; d.p.i. = days post-infection; CD = cell debris; SP = spore; the arrowhead indicates the cell debris; the arrow indicates the spore.

The Kaplan–Meier survival curve results showed that survival fitness was significantly reduced (*p* < 0.01) in the *N. ceranae*-infected *A. mellifera* (Figure 1A). Mature spores were observed from 5 to 20 d.p.i. in the homogenized midgut tissues of the *N. ceranae*-infected honey bees under microscopy; while only a few mature spores were observed at 5 d.p.i., the spore counts significantly increased from 10 to 20 d.p.i (Figure 1B,C). Molecular detection was performed to detect the infection process of *N. ceranae* on *A. mellifera*. Although the earliest time that mature spores were observed was at 5 d.p.i., the results showed that *N. ceranae* infection was first detected at 3 d.p.i. by PCR (Figure 1D).

### 3.2. Sequencing Data Summary

The sequencing data are summarized in Supplementary Table S2. A total of 1.68 million (M), 6.85 M, 1.79 M, 1.58 M, and 2.91 M raw PE reads were obtained from 0 (3 h), 5, 10, and 20 d.p.i. and mature *N. ceranae* spores. The number of clean reads was 1.44 M, 2.35 M, 1.57 M, 1.38 M, and 1.58 M for 0 (3 h), 5, 10, and 20 d.p.i. and mature *N. ceranae* spores, respectively. The mappability of each sample to the *N. ceranae* genome was 0%, 4%, 3%, 3%, and 12% for 0 (3 h), 5, 10, and 20 d.p.i. and mature *N. ceranae* spores, respectively. In addition, the newly emerged adult honey bees were confirmed to be free of *N. apis* and common honey bee viral infections (Supplementary Table S3). These mapped reads were subjected to further analysis of the expression of different genes.

### 3.3. DEG Analysis

**Figure 2:**
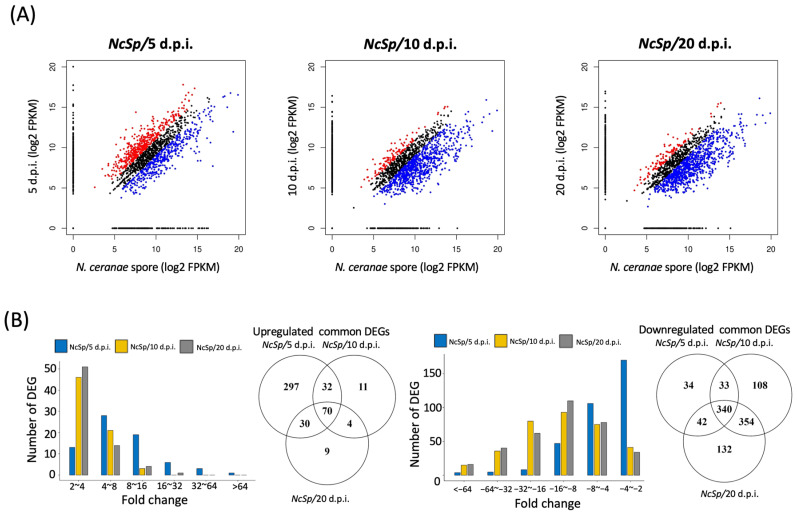
DEG and common gene identification. (**A**) Scatter plot of DEGs (red dots = upregulation and blue dots = downregulation); (**B**) Venn plot and fold-change number for the distribution of common DEGs.

For the DEG analysis, a total of 878, 952, and 981 genes were identified to have significant fold changes (fold change ≥ 2 or ≤−2) in NcSp/5 d.p.i., NcSp/10 d.p.i., and NcSp/20 d.p.i., respectively. Among these DEGs, 429, 117, and 113 genes were upregulated and 449, 835, and 868 genes were downregulated in NcSp/5 d.p.i., NcSp/10 d.p.i., and NcSp/20 d.p.i., respectively (Figure 2A; Table 1, Table 2, and Tables S4–S9). The common DEGs were selected from all three groups of common DEGs by set intersection, and stage-specific genes were defined as the genes that were only expressed at 5, 10, and 20 d.p.i., respectively (Table 3 and Table S10). There was a total of 70 upregulated and 340 downregulated common DEGs (Figure 2B; Table 1, Table 2, and Tables S4–S9), and the fold-change distribution at each infection stage is shown in Figure 2B. The results also showed that a greater upregulation of *N. ceranae* common DEGs and stage-specific genes was found in the initial infectious stage, whereas more genes of *N. ceranae* were suppressed in the late stage of infection (Figure 2). For the stage-specific genes, a total of 25, 71, and 70 genes were identified at each time post-infection (Supplementary Table S10), and the top 10 expression values (FPKM) of the stage-specific genes are summarized in Table 3.

### 3.4. Gene Ontology (GO) Enrichment Analysis

**Figure 3:**
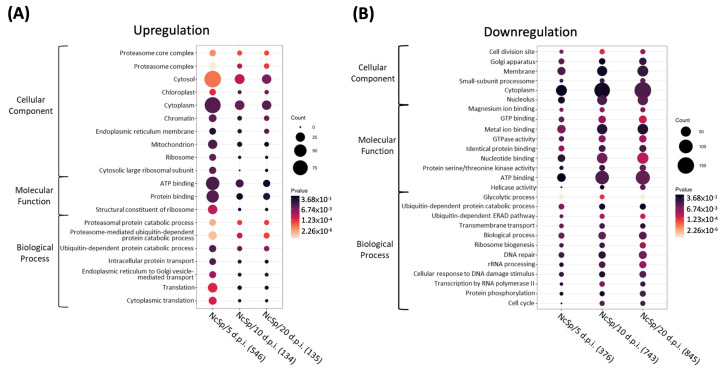
Dot plot of *N. ceranae* Gene Ontology (GO) term enrichment analysis, showing the enriched pathways with *p* < 0.05. (**A**) Upregulated DEGs; (**B**) downregulated DEGs. Each comparison is indicated at the bottom. The total number of regulated genes within the GO pathways selected on the dot plot are indicated in brackets. Colors indicate the p values from Fisher’s exact test, and the dot size is proportional to the number of DEGs and common DEGs in the pathway.

**Figure 4:**
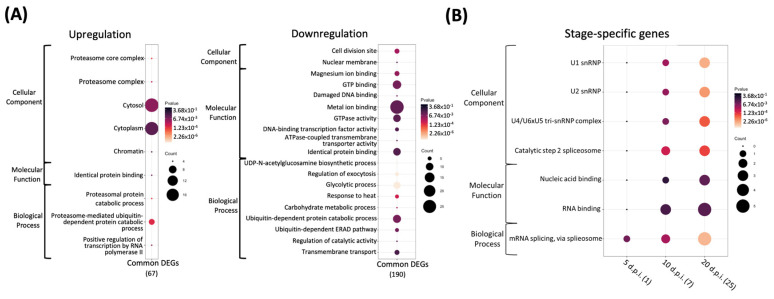
Dot plot of *N. ceranae* Gene Ontology (GO) term enrichment analysis, showing the enriched pathways with *p* < 0.05. (**A**) Core genes; (**B**) stage-specific genes. Each trial is indicated at the bottom; the total number of regulated genes within the GO pathways selected on the dot plot are indicated in brackets. Colors indicate the p values from Fisher’s exact test, and the dot size is proportional to the number of DEGs and common DEGs in the pathway.

The results of the GO annotation are shown in Figure 3. At NcSp/5 d.p.i., some genes belonging to GO pathways were significantly upregulated, including cytosol (GO:0005829), cytoplasm (GO:0005737), ATP binding (GO:0005524), protein binding (GO:0005515), and translation (GO:0006412), which were commonly found in NcSp/5 d.p.i., NcSp/10 d.p.i., and NcSp/20 d.p.i.; meanwhile, the genes involved in the aforementioned GO terms were reduced in number and downregulated in the NcSp/10 d.p.i. and NcSp/20 d.p.i. groups (Figure 3 and Supplementary Tables S11–S16). For the GO annotation of common DEGs, cytosol (GO:0005829) and cytoplasm (GO:0005737) were also found in the upregulated common DEGs group; however, metal ion binding (GO:0046872), GTP binding (GO:0005525), and the ubiquitin-dependent protein catabolic process (GO:0006511) showed high gene counts as observed in the downregulated common DEGs (Figure 4A and Supplementary Tables S11–S16). On the other hand, many stage-specific genes of *N. ceranae* were involved in pre-mRNA-associated pathways, including “RNA binding” (GO:0003723) and “mRNA splicing, via spliceosome” (GO:0000398) (Figure 4B).

### 3.5. KEGG Analysis

**Figure 5:**
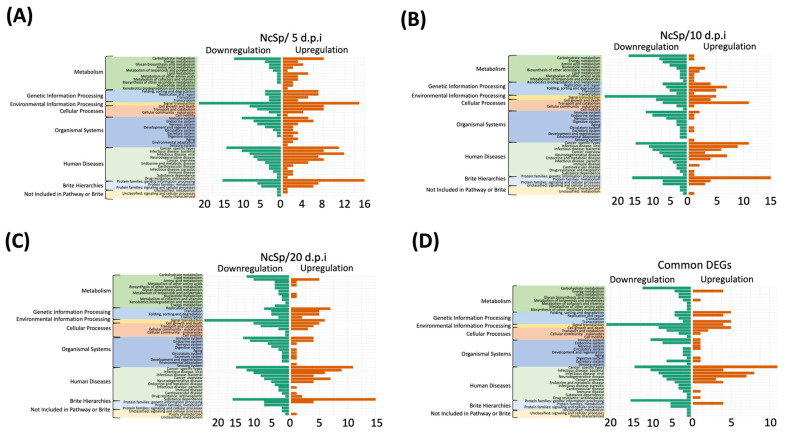
KEGG annotation of (**A**–**C**) DEGs and (**D**) common DEGs. The bar plots show the number of DEGs identified in the relative pathway based on the KEGG pathway database.

The results of the KEGG analysis show the pathway annotation of the DEGs and common DEGs at different stages and indicate the mechanisms of *N. ceranae* infection (Figure 5 and Supplementary Tables S17–S22). In the comparison of NcSp at 5 d.p.i., there were 211 pathways that included upregulated genes and 200 pathways that included downregulated genes (Figure 5A). Among these pathways, highly abundant *N. ceranae* DEGs were involved in the metabolism (KO:09100), environmental information processing (KO:09130), and organismal systems (KO:09150) categories, including the carbohydrate metabolism (KO:09101), signal transduction (KO:09132), and immune system (KO:09151) pathways (Figure 5A and Supplementary Tables S17 and S18). In the NcSp at 10 d.p.i. group, 112 pathways included upregulated genes and 1224 pathways included downregulated genes. Among these pathways, three categories, including the metabolism pathway (KO:09100), human diseases (KO:09160), and environmental information processing (KO:09130) were the most mapped, and they contained the pathways of carbohydrate metabolism (KO:09101) and signal transduction (KO:09132) (Figure 5B; Supplementary Tables S19 and S20). For the NcSp/20 d.p.i. group, there were 129 pathways that included upregulated genes and 267 pathways that included downregulated genes, and the pathway categories that highly regulated genes belonged to, including metabolism pathways (KO:09100), environmental information processing (KO:09130), human diseases (KO:09160), and organismal systems (KO:09150), were identified. These pathways also included carbohydrate metabolism (KO:09101) and signal transduction (KO:09132) (Figure 5C; Supplementary Tables S21 and S22).

The KEGG analysis results of the common DEGs were similar to those of the DEGs (Figure 5D; Supplementary Tables S23 and S24). A total of 80 pathways included upregulated genes and 199 pathways included downregulated genes during *N. ceranae* infection (Figure 5D). A high number of downregulated common DEGs were involved in the pathways of signal transduction (KO:09132); protein families: genetic information processing (KO:09182) and carbohydrate metabolism (KO:09101) within the environmental information process (KO:09130); brite hierarchies (KO:09180); and metabolism (KO:09100) categories.

### 3.6. Validation

**Figure 6:**
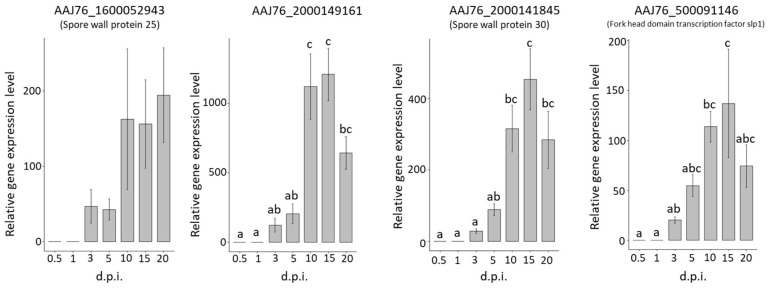
Validation of *N. ceranae* gene expression in upregulated common DEGs during *N. ceranae* infection. The bar plots show the relative gene expression levels of different common DEGs at different d.p.i. Different letters indicate significant differences (*p* < 0.05).

Four highly upregulated common DEGs (AAJ76_1600052943, AAJ76_2000149161, AAJ76_2000141845, and AAJ76_500091146) and three highly expressed stage-specific genes (AAJ76_5000026485, AAJ76_2400020190, and AAJ76_2300035467) at each time post-infection were selected to validate the RNA-seq results during infection by *N. ceranae*. The results showed that all of the upregulated common DEGs increased at 3 d.p.i. and reached a high peak at 15 to 20 d.p.i., while no or a low abundance of gene expression was detected at 0.5 to 1 d.p.i. (Figure 6). The validation result matched the detection of *N. ceranae* infection, which was detectable at 3 d.p.i. (Figure 1D), suggesting that *N. ceranae* begins the infection process and initiates gene expression at 3 d.p.i. In addition, the upregulated common DEGs, reaching a high peak at 15–20 d.p.i., also showed coordination with the increasing spore count of *N. ceranae* (Figure 1C).

**Figure 7:**
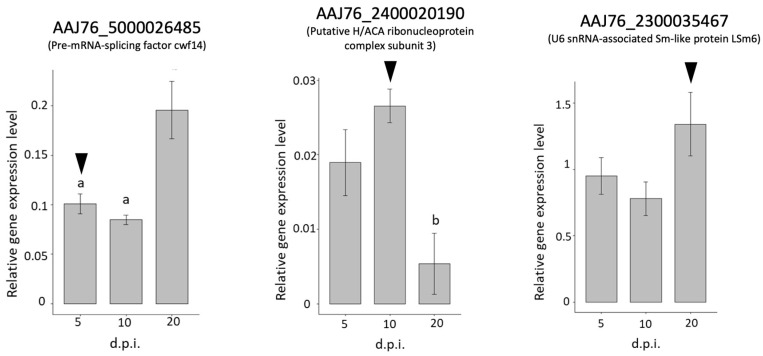
Validation of *N. ceranae* gene expression in upregulated stage-specific genes during *N. ceranae* infection. The bar plots show the relative gene expression levels of different stage-specific genes at different d.p.i. The black arrowheads indicate the time point at which the highest expression level of the gene was predicted based on RNA-seq data. Different letters indicate significant differences (*p* < 0.05).

For the stage-specific gene validations, the stage-specific genes were only expressed at specific times post-infection, while the gene expression of AAJ76_5000026485, AAJ76_2400020190, and AAJ76_2300035467 was detected at all times post-infection, including in mature spores (Figure 7). However, the results of three stage-specific genes showed higher expression levels at 5, 10, and 20 d.p.i., revealing a similar expression trend as the transcriptomic prediction (Figure 7).

## 4. Discussion

The pattern of *N. ceranae* infection in this study was similar to a previous report that *N. ceranae* was detectable in an early infection stage at 3 d.p.i., while mature spores were only observed at 5 d.p.i. [18,49]. Negative impacts on the survival fitness of *N. ceranae*-infected honey bees have been reported, and they explain the changes to immune suppression, nutritional deficiencies, and hormone regulation [18,50,51,52]. These data indicate that infected *N. ceranae* might begin to affect and have consistent negative effects on honey bees at earlier infectious stages. Altogether, these data reveal that *N. ceranae* infections can be detected as early as 3 d.p.i. and the infection level dramatically increases from 10 to 20 d.p.i.

The gene expression profiling of *N. ceranae* was described at the transcriptomic level in this study. The results indicated that a greater upregulation of *N. ceranae’s* common and stage-specific genes was found in the initial infectious stage, whereas more genes of *N. ceranae* were suppressed in the late stage of infection. This tendency was similar to the study of *N. ceranae* gene expression in *Apis cerana,* suggesting that the *N. ceranae* genes were expressed at an early infection stage, followed by the gene expression profile of *N. ceranae* switching from the merogony to sporogony stages [53]. Microsporidia lack mitochondria, and the loss of most metabolic pathways might involve an alteration of gene expression during the course of infection; therefore, host-generated ATP would be imported to support their metabolism [54,55]. Several highly expressed stage-specific genes (AAJ76_5000026485, AAJ76_2400020190, AAJ76_2300035467, and AAJ76_600074061) were identified as pre-mRNA splicing factors, indicating that post-transcriptional regulation was continually activated during *N. ceranae* infection.

For the GO annotation of common DEGs, cytosol (GO:0005829) and cytoplasm (GO:0005737) were also found in the upregulated common DEG group. The cytoplasm mainly comprises microsporidia, containing many ribosomes in a regular array [56]. During the replication of sporonts, division by binary fission produces two sporoblasts and infects the hosts through karyokinesis and cytokinesis [2]. Therefore, the upregulation of genes belonging to the cytoplasm with corresponding GO terms might contribute to the early propagation of *N. ceranae*. At the initiation of microsporidial infection, sporoplasm was injected into the cytoplasm of the host cell by the germination of the polar tube [2]. Because of the lack of genes for many metabolic pathways in the microsporidia, the microsporidia acquire nutrients from the host. Microsporidia in contact with the host cytoplasm could provide more opportunities to export cytotoxic compounds across the plasma membrane as an alternative to compensate for development [57]. In addition, upregulated genes belonging to the ATP-binding (GO:0005524) pathway were found in the early stage of *N. ceranae* infection (NcSp/5 d.p.i.). In the ATP-binding (GO:0005524) pathway, the transmembrane protein superfamily of microsporidia, which includes ATP-binding cassette (ABC) transporters, plays an important role in substrate transportation and pathogen development, indicating that energy transport is abundant in this stage [57].

It was noted that metal ion binding (GO:0046872), GTP binding (GO:0005525), and the ubiquitin-dependent protein catabolic process (GO:0006511) showed high gene counts as observed in the downregulated common DEGs. Metal ions are vital elements for proper folding and the ribozyme catalysis of nucleic acids [58,59,60]. GTP-binding proteins regulate cellular processes for multiple protein biosynthesis and intracellular membrane traffic [61]. The role of the ubiquitin–proteasome pathway has been reported to be the selective degradation of soluble cellular proteins under most conditions [62]. The downregulation of the genes belonging to the GO term indicate the requirement of nucleotide and protein bases from host cells in *N. ceranae* infections. In contrast, many stage-specific genes of *N. ceranae* were involved in pre-mRNA-associated pathways, including “RNA binding” (GO:0003723) and “mRNA splicing, via spliceosome” (GO:0000398), suggesting the impacts on the transcriptional level for *N. ceranae* infection.

Based on the result of KEGG pathway analysis on DEGs, the categories of metabolism and environmental information processing included a high number of downregulated genes. Moreover, in the pathways of metabolism, the “carbohydrate metabolism” included a high number of downregulated genes at each infection stage. It has been reported that *Nosema* acquires carbohydrates from the epithelial cells of the honey bee gut lining, leading to a series of metabolic changes in the host [3,20]; therefore, this point was also proven by the downregulation of genes belonging to “carbohydrate metabolism”.

Instead of mitochondria, microsporidia contain a genome-less organelle called a mitosome, which encodes only 20 mitosomal proteins in microsporidia, indicating the loss of function of the mitosome organ compared to that of normal mitochondria in the yeast *Saccharomyces cerevisiae* [32,63]; therefore, the mature spores could still maintain basic metabolism by their own system. In the beginning of infection, genes belonging to the “folding, sorting, and degradation (K09123)” and “replication and repair (K09124)” pathways in the genetic information processing (K09120) categories, the cellular processes pathway (KO:09140) of “cell growth and death” (K09143), and the environmental information processing pathway (KO:09130) of “signal transduction” (K09132) were highly upregulated in the NcSp/5 d.p.i. group. Meanwhile, the genes involved in these pathways were downregulated at a late stage of *N. ceranae* infection (NcSp/10 d.p.i. and NcSp/20 d.p.i. groups), suggesting that the merogony stage needs to initiate more functions of genetic and information processes than mature spores to facilitate the propagation of *N. ceranae* and maintain the activities of infection. Lastly, from our data, the downregulated genes involved in the pathway of carbohydrate metabolism suggest that *N. ceranae* takes energy from honey bees during infection [8].

The validation of four highly upregulated common DEGs and three highly expressed stage-specific genes at each time post-infection showed similar tendencies to those of the DEG predictions. The AAJ76_1600052943 and AAJ76_2000141845 showed a functional homology to the spore wall proteins SWP25 and SWP30, respectively. In *Nosema bombycis*, the two spore wall proteins have been characterized as an endosporal protein (SWP30) and an exosporal protein (SWP32) [64,65]. These two spore wall proteins bind to the chitin component of *N. bombycis*, implying that these proteins are the structural support proteins in the spore wall. SWP25 was first found in *N. bombycis*; the localization of SWP25 is the endospore, and this protein possesses a signal peptide and a heparin-binding motif. In addition, SWP25 is critical for host-to-host transmission and persistence in the environment [66]. Furthermore, SWP25 has been reported to be related to spore wall integrity and germination, and recent research has shown that the induction of SWP25 expression after binding with haematopoietin may change the integrity of pathogens and affect the clearance of immune cells or the transmission of pathogens in haemolymph [65,67]. Therefore, the upregulation of AAJ76_1600052943 and AAJ76_2000141845 might also play roles similar to those mentioned above to facilitate spore germination and mature spore development. The highly expressed SPW was consistent with our previous study on *N. ceranae* gene expression in *A. mellifera* [33] and the research on the *N. ceranae* gene expression in *A. cerana,* suggesting a significant role for SPW during *N. ceranae* infection [53].

AAJ76_2000149161 was mapped to a DNA-dependent protein kinase catalytic subunit (DNA-PKcs) by SWISS-MODEL [68]. DNA-PKcs is a key member of the phosphatidylinositol-3 kinase-like (PIKK) family of protein kinases and plays an essential role in the repair of DNA double-strand breaks by nonhomologous end joining (NHEJ) [69]. The upregulation of this gene might contribute to the relative function of *N. ceranae* in using host DNA fragments for its replication. AAJ76_500091146 is described as a forkhead hnf3 transcription factor based on protein functional prediction. HNF-3/FKH genes are a large family of transcriptional activators, and all forkhead proteins contain a highly conserved DNA binding domain and play numerous regulatory roles in eukaryotes. At least four genes in yeast are involved in a wide range of biological functions, such as apoptosis, cell cycle, immunity, development, growth, stress resistance, metabolism, and reproduction [70,71]. Based on the above functions, the upregulated AAJ76_500091146 might be involved in multiple regulatory roles in the development and growth of *N. ceranae*.

Interestingly, the functional prediction of all three stage-specific genes indicated post-transcriptional modification. AAJ76_5000026485 is a functional homologue of the pre-mRNA-splicing factor cwf14, which is a type of spliceosome that catalyzes the excision of introns from pre-mRNA [72]. A study from yeast showed that cwf14 functions in the regulation of cell growth and mRNA splicing and is also a key factor regulating cell wall biogenesis [73]. The function of AAJ76_2400020190 was found to match that of the putative H/ACA ribonucleoprotein complex subunit 3, which is known as a small nuclear RNP (snRNP). snRNP predominantly guides the site-directed pseudouridylation of target RNAs, processes ribosomal RNA, and stabilizes telomerase RNA [74]. Moreover, the function of H/ACA ribonucleoproteins is essential for ribosome biogenesis, pre-mRNA splicing, and telomere maintenance [74]. AAJ76_2300035467 is functionally similar to U6 snRNA-associated Sm-like protein LSm6. The Sm proteins are RNA–protein complex proteins, and LSm6 is an Sm-like protein (LSM) in *S. cerevisiae*. A study of *S. cerevisiae* suggested that LSm6 is associated with U6 snRNA and is required for the maintenance of normal U6 snRNA levels and for pre-mRNA splicing [75]. In summary, the primary transcription products (pre-mRNAs) undergo a variety of post-transcriptional modifications to create functional mRNAs [76]. These three stage-specific genes were identified at different infection stages, and all associations with pre-mRNA splicing suggest that they are highly involved in the activation of post-transcriptional modifications of *N. ceranae* at each infectious stage.

## 5. Conclusions

In this study, the transcriptomic analysis results indicated alterations in the *N. ceranae* gene expression status during *A. mellifera* infection. The identification of the common and stage-specific genes expressed during the infection process revealed the interaction between *N. ceranae* and *A. mellifera*. Downregulated genes during the infection, especially at a late period of infection, were found. Regarding RT–qPCR validation, the common DEGs showed higher expression levels from 5 d.p.i. to 20 d.p.i., indicating the importance of common DEGs. In our study, we attempted to identify the DEGs resulting from gene expression profiling based on transcriptomic analysis and proposed the common and stage-specific genes of *N. ceranae* during its infection of *A. mellifera*. Our data also describe the relatively comprehensive gene expression of the *N. ceranae* infection stage and analyze the details during infection, thus providing basic information on the *N. ceranae* mechanism during infection and furthering the application, including the early detection of nosemosis.

## Figures and Tables

**Table 1:** List of upregulated common DEGs with FPKM values > 1000 at different times post-infection.

No.	Gene ID	FPKM (Mature Spore/5 d.p.i/10 d.p.i/20 d.p.i.)	Fold Change			Gene Type	Protein ID	Length (aa)	Protein Name		E-Value	KO Annotation
			NcSp/5 d.p.i.	NcSp/10 d.p.i.	NcSp/20 d.p.i.				NCBI	EMBL-EBI		
1 *	AAJ76_1600052943	15,626.3/115,903/34,433.6/47165.2	7.417124574	2.203565835	3.018307934	Protein coding	XP_024331363.1	265	Hypothetical protein	Spore wall protein 25 (SWP25_NOSB1)	1.50 × 10^−13^	-
2 *	AAJ76_2000149161	12,535.3/91,333/34,160.1/43545.1	7.286065679	2.725120453	3.473788695	Protein coding	XP_024332331.1	263	Hypothetical protein	-	-	-
3 *	AAJ76_2000141845	11,759.8/65,562.7/29,751.5/34741.6	5.575147599	2.529916904	2.95426655	Protein coding	XP_024332326.1	341	Hypothetical protein	Spore wall protein 30 (SWP32_NOSB)	6.00 × 10^−13^	-
4	AAJ76_1600051435	189.533/21,504.1/1488.81/3201.29	113.4584304	7.855156397	16.89038139	Protein coding	XP_024331362.1	286	Hypothetical protein	Spore wall protein 25 (SWP25_NOSB)	1.50 × 10^−6^	-
5	AAJ76_2300022418	6875.45/16,903.7/17,987.8/19,037.2	2.458553927	2.616231128	2.768875144	Protein coding	XP_024331106.1	198	Hypothetical protein	WD repeat-containing protein 48 homolog (WDR48_DICDI)	3.30 × 10^−5^	-
6	AAJ76_8000115509	783.185/14,628.1/1618.08/1750.26	18.67776596	2.066013956	2.234791131	Protein coding	XP_024331780.1	331	Hypothetical protein	Radixin (RADI_PIG)	9.70 × 10^−7^	-
7	AAJ76_500093385	836.136/12,300.3/3753.56/3436.68	14.71096519	4.48917395	4.110203999	Protein coding	XP_024332003.1	166	Hypothetical protein	-	-	-
8	AAJ76_500085842	830.632/8069.28/1893.56/1693.65	9.714636403	2.279662102	2.038997491	Protein coding	XP_024331997.1	187	Hypothetical protein	-	-	-
9	AAJ76_700014852	292.943/6718.83/2035.24/2486.37	22.93565176	6.947577391	8.487560776	Protein coding	XP_024331793.1	154	Ubiquitin-conjugating enzyme e2 e2	Ubiquitin-conjugating enzyme E2 E2 (UB2E2_MOUSE)	1.10 × 10^−60^	K20217
10 *	AAJ76_500091146	142.197/5518/1477.09/1142.36	38.80552048	10.38766869	8.033618765	Protein coding	XP_024332001.1	198	Forkhead hnf3 transcription factor	Fork head domain transcription factor slp1 (SLP1_DROME)	6.30 × 10^−25^	K09399
11	AAJ76_1500044498	794.305/5221.61/2185.54/1886.35	6.573811096	2.75152226	2.374839476	Protein coding	XP_024331404.1	233	Proteasome subunit alpha type-5	Probable proteasome subunit alpha type-5 (PSA5_ENCCU)	8.10 × 10^−98^	K02729
12	AAJ76_1040006841	612.16/4981.59/1332.9/1251.91	8.137750225	2.177375154	2.04506968	Protein coding	XP_024329929.1	708	cdc48-like aaa ATPase	-	-	K13525
13	AAJ76_7900011594	530.277/3735.63/2918.4/1222.32	7.044659616	5.503540416	2.305069101	Protein coding	XP_024330110.1	199	Peptidyl-prolyl cis-trans isomerase	Peptidyl-prolyl cis-trans isomerase B (PPIB_RAT)	1.00 × 10^−40^	K03768
14	AAJ76_5600010148	487.495/2612.36/2107.93/1300.2	5.358762956	4.324010421	2.667114941	Protein coding	XP_024330347.1	149	Signal peptidase complex subunit 3	Probable signal peptidase complex subunit 3 (SPCS3_CAEEL)	3.30 × 10^−5^	K12948
15	AAJ76_2400023015	423.143/2460.52/1559.48/1320.58	5.81488234	3.685473539	3.120873758	Protein coding	XP_024331065.1	229	Proteasome subunit alpha type-2-a	Proteasome subunit alpha type-2 (PSA2_ORYSJ)	3.40 × 10^−36^	K02726
16	AAJ76_3500034447	417.662/1822.04/1577.38/2178.81	4.362484484	3.776683799	5.21667299	Protein coding	XP_024330780.1	205	20s proteasome subunit	Proteasome subunit beta type-3 (PSB3_DICDI)	1.50 × 10^−47^	K02735
17	AAJ76_1100054231	704.144/1773.09/1670.11/1547.41	2.518072811	2.371828999	2.197586572	Protein coding	XP_024331572.1	206	Udp-glucose pyrophosphorylase	-	-	-

* = selected gene for validation; FPKM = fragment per kilobase per million; d.p.i. = days post-infection; aa = amino acid; NCBI = National Center for Biotechnology Information; EMBL-EBI = the European Molecular Biology Laboratory European Bioinformatics Institute.

**Table 2:** List of downregulated common DEGs with FPKM values > 1000 at different times post-infection.

No.	Gene ID	FPKM (Mature Spore/5 d.p.i/10 d.p.i/20 d.p.i.)		Fold Change		Gene Type	Protein ID	Length (aa)	Protein Name		E-Value	KO Annotation
			NcSp/5 d.p.i.	NcSp/10 d.p.i.	NcSp/20 d.p.i.				NCBI	EMBL-EBI		
1	AAJ76_630009567	398,502/32947.1/61,754.1/70,676.1	−12.0951772	−6.453044615	−5.6384163	Protein coding	XP_024330265.1	107	Hypothetical protein	-	-	K09140
2	AAJ76_2000146453	522,948/111,302/18,531.6/16,522.3	−4.698434055	−28.21909861	−31.65101806	Protein coding	XP_024332329.1	183	Hypothetical protein	Cilia- and flagella-associated protein 251 (CF251_MOUSE)	2.00 × 10^−7^	-
3	AAJ76_1600023921	978,848/95,231.2/24,913.3/19,641	−10.27865792	−39.29026518	−49.83681619	Protein coding	XP_024331347.1	267	Hypothetical protein	Nuclear protein 58 (NOP58_DICDI)	2.40 × 10^−20^	-
4	AAJ76_1900025375	321,105/84,821.6/14,069.3/17,252.2	−3.785647288	−22.8230548	−18.61237171	Protein coding	XP_024331241.1	275	Polar tube protein 2	Polar tube protein 2 (PTP2_ENCCU)	2.60 × 10^−6^	-
5	AAJ76_1900028347	282,631/79,179.8/8975.75/7515.33	−3.569495047	−31.4884317	−37.60727843	Protein coding	XP_024331242.1	446	Hypothetical protein	Proline-rich protein 2 (PRP2_MOUSE)	3.10 × 10^−22^	K10844
6	AAJ76_1200007387	104,357/36,502.7/9664.27/6697.23	−2.858899688	−10.79820795	−15.58212405	Protein coding	XP_024329835.1	464	Elongation factor 1	Elongation factor 1-alpha (EF1A_ENCCU)	1.60 × 10^−168^	K03857
7	AAJ76_6000015274	135,745/34,449.1/7043/6780.66	−3.940448921	−19.27367797	−20.01944398	Protein coding	XP_024330295.1	236	Hypothetical protein	-	-	-
8	AAJ76_4500017931	80,571.7/30,587.7/9618.05/9332.46	−2.634118045	−8.377152866	−8.633466829	Protein coding	XP_024330533.1	239	Hypothetical protein	-	-	-
9	AAJ76_210002401	59,830.8/26,523.1/6006.5/3556.09	−2.255801308	−9.960995968	−16.82494636	Protein coding	XP_024331167.1	678	Heat shock protein 70	-	-	-
10	AAJ76_130006800	105,634/24,439/2415.33/1928.25	−4.322332328	−43.73480633	−54.78199877	Protein coding	XP_024331464.1	204	Hypothetical protein	-	-	-
11	AAJ76_3100015858	54,826.6/22,173.9/2287.82/2631.64	−2.47256766	−23.96455092	−20.83359639	Protein coding	XP_024330869.1	338	Hypothetical protein	Uncharacterized protein ECU05_0110 (Y511_ENCCU)	1.60 × 10^−7^	-
12	AAJ76_420004908	91,596.5/21,857.3/5018.44/4703.25	−4.190667331	−18.25196643	−19.47511578	Protein coding	XP_024330583.1	221	Hypothetical protein	Spore wall protein ECU02_0150 (Y215_ENCCU)	6.70 × 10^−25^	K01689
13	AAJ76_2700043085	39,025.5/19,267.6/3836.51/3059.65	−2.02544653	−10.17213071	−12.7549028	Protein coding	XP_024330997.1	156	Hypothetical protein	-	-	-
14	AAJ76_1200044876	84,245.8/18,063.1/3850.84/6236.68	−4.663974821	−21.87725765	−13.50807323	Protein coding	XP_024331532.1	222	Spore wall protein precursor	Spore wall protein 26 (SWP26_NOSB1)	5.50 × 10^−29^	-
15	AAJ76_4700024603	67,259.6/16,069.1/3500.31/3049.83	−4.185645126	−19.2153853	−22.05356525	Protein coding	XP_024330499.1	284	ABC-type multidrug transport ATPase and permease component	Spore wall protein 7 (SWP7_NOSB1)	2.80 × 10^−100^	-
16	AAJ76_6600013598	34,656.4/16,003.4/2672.38/2821.32	−2.165559782	−12.96832278	−12.28375723	Protein coding	XP_024330229.1	317	Hypothetical protein	Transcription initiation factor TFIID subunit 3 (TAF3_CHICK)	3.10 × 10^−6^	-
17	AAJ76_1400036761	95,516.2/14,664.3/5710.68/3838.4	−6.513531706	−16.72587754	−24.88440523	Protein coding	XP_024331438.1	226	Hypothetical protein	-	-	-
18	AAJ76_2400032169	38,208.9/13,393.2/2471.53/3059.12	−2.852862075	−15.45958836	−12.49015954	Protein coding	XP_024331074.1	131	Hypothetical protein	Histone-lysine N-methyltransferase, H3 lysine-79 specific (9DOT1L_DICDI)	1.10 × 10^−6^	K07178
19	AAJ76_5300022630	39,320.1/12,093.2/3489.63/3466.58	−3.251442701	−11.267691	−11.34260414	Protein coding	XP_024330400.1	295	Hydrolase of the alpha beta-hydrolase	Uncharacterized protein ECU07_0920 (Y792_ENCCU)	1.60 × 10^−37^	-
20	AAJ76_1000065899	96,438/11,363/4552.52/4869.86	−8.487031311	−21.18351365	−19.80303982	Protein coding	XP_024331635.1	216	Hypothetical protein	-	-	-
21	AAJ76_4000112883	117,599/9836.2/2044.46/1175.49	−11.95572738	−57.52072681	−100.0425551	Protein coding	XP_024332099.1	327	glyceraldehyde-3-phosphate dehydrogenase	Glyceraldehyde-3-phosphate dehydrogenase (G3P_CUTCU)	2.10 × 10^−120^	K00134
22	AAJ76_5000109749	21,356.7/9085.52/2351.82/2527.34	−2.350634122	−9.080929654	−8.450285003	Protein coding	XP_024332015.1	229	Spore wall protein 12	Spore wall protein 12 (SWP12_NOSB1)	2.20 × 10^−76^	-
23	AAJ76_2300036398	21,838.9/8533.66/3482.57/2535.92	−2.559141644	−6.270932541	−8.611771259	Protein coding	XP_024331119.1	380	Spore wall protein 9	Spore wall protein 9 (SWP9_NOSB1)	2.00 × 10^−39^	K07955
24	AAJ76_2700031779	26,552.2/7739.67/1271.69/1831.42	−3.430669148	−20.87942389	−14.498181	Protein coding	XP_024330986.1	167	Hypothetical protein	-	-	-
25	AAJ76_6600019728	28,729.1/5107.81/2222.85/3656.61	−5.624520074	−12.9244412	−7.856735542	Protein coding	XP_024330233.1	154	Prenylated Rab acceptor 1-like protein	Uncharacterized membrane protein ECU02_1470 (Y2E7_ENCCU)	3.70 × 10^−16^	K11838
26	AAJ76_420004134	18,934/5107.54/1904.29/1739.95	−3.707071651	−9.942853842	−10.88191078	Protein coding	XP_024330582.1	219	Ran-specific GTPase-activating protein 1	Ran-specific GTPase-activating protein 1 (YRB1_ENCCU)	3.70 × 10^−41^	-
27	AAJ76_200059848	19,359.4/4708.45/1848.74/1879.72	−4.111628734	−10.47167266	−10.29905455	Protein coding	XP_024332275.1	250	Hypothetical protein	Gelsolin-related protein of 125 kDa (GNRA_DICDI)	3.90 × 10^−5^	-
28	AAJ76_1600068502	14,848.7/4309.26/2652.73/2834.43	−3.445778582	−5.597528622	−5.238704187	Protein coding	XP_024331374.1	122	ccaat binding transcription factor subunit a	Nuclear transcription factor Y subunit B-5 (NFYB5_ARATH)	1.20 × 10^−24^	-
29	AAJ76_1100066671	659,735/4001.88/4268.18/8495.99	−164.8568473	−154.570372	−77.65247484	Protein coding	XP_024331582.1	132	Hypothetical protein	Protein DR_1172 (UB72_DEIRA)	8.90 × 10^−15^	K11204
30	AAJ76_1000142585	18,723.8/3995.19/1946.34/2392.26	−4.686594587	−9.620020711	−7.826840255	Protein coding	XP_024332442.1	268	Septin	Cell division control protein 11 (CDC11_ENCCU)	4.30 × 10^−98^	K01151
31	AAJ76_280006733	32,568.2/3946.19/1832.71/1589.86	−8.253064983	−17.77059399	−20.48491498	Protein coding	XP_024330938.1	201	Hypothetical protein	-	-	-
32	AAJ76_1700062503	19,005.2/2947.71/7507.76/2855.59	−6.447411209	−2.531407907	−6.655421327	non coding RNA	-	-	23S ribosomal RNA	-	-	-
33	AAJ76_710008572	22,045.4/2941.14/1179.25/1501.71	−7.495527438	−18.69447434	−14.68010111	Protein coding	XP_024330184.1	190	Hypothetical protein	-	-	K00162
34	AAJ76_100008081	73,487.3/2295.2/1218.66/2315.53	−32.01774949	−60.30141705	−31.73669519	Protein coding	XP_024331599.1	209	Hypothetical protein	-	-	-
35	AAJ76_1000165053	8466.55/1740.59/1290.67/1686.56	−4.864191406	−6.559791681	−5.020009172	Protein coding	XP_024332456.1	347	Septin-like protein	Cell division control protein 10 (CDC10_ENCCU)	8.30 × 10^−110^	-
36	AAJ76_1000016205	97,815.6/1570.42/2985.17/2356.31	−62.28660354	−32.76718881	−41.51209968	Protein coding	XP_024331606.1	186	Hypothetical protein	-	-	-

FPKM = fragment per kilobase per million; d.p.i. = days post-infection; aa = amino acid; NCBI = National Center for Biotechnology Information; EMBL-EBI = the European Molecular Biology Laboratory European Bioinformatics Institute.

**Table 3:** List of the top 10 ranked stage-specific genes at different times post-infection.

Stage	No.	Gene ID	FPKM	Protein ID	Length (aa)	Protein Name		E-Value	KO Annotation
						NCBI	EMBL-EBI		
5 d.p.i	1 *	AAJ76_5000026485	2388.86	XP_024330451.1	116	g10 protein	Pre-mRNA-splicing factor cwf14 (CWF14_SCHPO)	7.40 × 10^−27^	-
	2	AAJ76_500072312	1067.07	XP_024331989.1	149	Hypothetical protein	-	-	-
	3	AAJ76_4500025263	987.901	XP_024330536.1	176	Hypothetical protein	-	-	-
	4	AAJ76_500090033	754.257	XP_024332000.1	164	Nuclear essential protein 1	Ribosomal RNA small subunit methyltransferase mra1 (NEP1_SCHPO)	8.40 × 10^−11^	K14568
	5	AAJ76_4800030660	679.592	XP_024330487.1	132	Hypothetical protein	-	-	-
	6	AAJ76_2700033485	549.13	XP_024330988.1	174	Hypothetical protein	-	-	-
	7	AAJ76_2200018137	404.002	XP_024331140.1	145	Deoxycytidylate deaminase	tRNA-specific adenosine deaminase 2 (ADAT2_XENTR)	8.60 × 10^−22^	K15441
	8	AAJ76_900062461	401.31	XP_024331679.1	148	Bis (5 -adenosyl)-triphosphatase-like protein	Bis(5’-adenosyl)-triphosphatase (FHIT_DICDI)	1.00 × 10^−25^	K01522
	9	AAJ76_1000019447	326.831	XP_024331608.1	187	Hypothetical protein	-	-	-
	10	AAJ76_1400060351	208.221	XP_024331456.1	199	Hypothetical protein	Putative chaperone PpiD (PPID_BUCBP)	1.60 × 10^−5^	-
10 d.p.i	1	AAJ76_2850002256	5462.22	XP_024329453.1	41	Hypothetical protein	-	-	-
	2	AAJ76_2900015300	3735.49	XP_024330918.1	41	Hypothetical protein	-	-	-
	3	AAJ76_1100010265	3263.98	XP_024331553.1	43	Hypothetical protein	-	-	-
	4 *	AAJ76_2400020190	2691.71	XP_024331062.1	53	H aca ribonucleoprotein complex subunit 3	Putative H/ACA ribonucleoprotein complex subunit 3 (NOP10_CAEEL)	8.80 × 10^−11^	-
	5	AAJ76_900027941	1915.56	XP_024331657.1	63	Hypothetical protein	-	-	-
	6	AAJ76_1100054191	1914.4	XP_024331571.1	56	Hypothetical protein	Exportin-T (XPOT_DICDI)	4.80 × 10^−4^	-
	7	AAJ76_2160003	1472.66	XP_024329567.1	62	Nol1 nol2 sun tRNA rRNA cytosine-c5-methylase	tRNA (cytosine(48)-C(5))-methyltransferase (TRM4_METJA)	2.80 × 10^−12^	K14835
	8	AAJ76_1120001085	1441.18	XP_024329876.1	52	Hypothetical protein	-	-	-
	9	AAJ76_3600023778	1344.19	XP_024330748.1	63	Hypothetical protein	-	-	-
	10	AAJ76_1000166313	1281.3	XP_024332457.1	64	Hypothetical protein	-	-	-
20 d.p.i	1	AAJ76_3000132534	5797.14	XP_024332206.1	46	Hypothetical protein	-	-	-
	2 *	AAJ76_2300035467	5519.29	XP_024331118.1	67	Hypothetical protein	U6 snRNA-associated Sm-like protein LSm6 (LSM6_SCHPO)	8.70 × 10^−7^	-
	3	AAJ76_6100016064	2662.52	XP_024330287.1	53	Hypothetical protein	-	-	-
	4	AAJ76_600074061	1545.98	XP_024331899.1	87	Zinc finger domain-containing protein	Pre-mRNA-splicing factor CWC24 (CWC24_KLULA)	2.70 × 10^−10^	K13127
	5	AAJ76_347000188	1394.2	XP_024329388.1	62	Hypothetical protein	-	-	-
	6	AAJ76_1720005146	1258.07	XP_024329669.1	61	Calcium-binding protein of the recoverin subfamily	Neuronal calcium sensor 1 (NCS1_LYMST)	3.60 × 10^−9^	K19932
	7	AAJ76_400028941	1057.47	XP_024332054.1	86	Ubiquitin-related modifier 1	Ubiquitin-related modifier 1 (URM1_LACBS)	5.40 × 10^−7^	-
	8	AAJ76_230009864	789.777	XP_024331097.1	82	Hypothetical protein	-	-	-
	9	AAJ76_590008095	769.682	XP_024330308.1	84	Hypothetical protein	-	-	-
	10	AAJ76_610001	724.872	XP_024330279.1	69	Hypothetical protein	-	-	-

d.p.i.= days post-infection; * = selected gene for validation; FPKM = fragment per kilobase per million; aa = amino acid; NCBI = National Center for Biotechnology Information; EMBL-EBI = the European Molecular Biology Laboratory European Bioinformatics Institute.

## Data Availability

The RNA-Seq data were deposited in NCBI with BioProject ID: PRJNA814859.

## Supplementary Materials

The following supporting information can be downloaded at: https://www.mdpi.com/article/10.3390/insects13080716/s1, Figures S1 and S2; Tables S1–S24. Figure S1. The detection of *Nosema* spp. of newly emerged adult honey bees by multiplex PCR before experiment. The information of primer sets were listed in Supplementary Table S1; Figure S2. The pipeline of transcriptome analysis. The commend lines and analysis purposes are noted pairwise the item. DEG = differential expressed gene; FPKM = fragment per kilobase per million; GO = gene ontology; KEGG = Kyoto Encyclopedia of Genes and Genomes; Table S1. Primer sets list; Table S2. RNA sequencing summary; Table S3. Transcriptomic data for honey bee infected with Nosema ceranae were screened by mapping to the genomes of Nosema apis and 6 honey bee infecting viruses; Table S4. Up-regulated DEG list of NcSp/5 d.p.i. comparison group; Table S5. Down-regulated DEG list of NcSp/5 d.p.i. comparison group; Table S6. Up-regulated DEG list of NcSp/10 d.p.i. comparison group; Table S7. Down-regulated DEG list of NcSp/10 d.p.i. comparison group; Table S8. Up-regulated DEG list of NcSp/20 d.p.i. comparison group; Table S9. Down-regulated DEG list of NcSp/20 d.p.i. comparison group; Table S10. Non-core genes list at each time post infection; Table S11. Up-regulated GO list of NcSp/5 d.p.i. comparison group; Table S12. Down-regulated GO list of NcSp/5 d.p.i. comparison group; Table S13. Up-regulated GO list of NcSp/10 d.p.i. comparison group; Table S14. Down-regulated GO list of NcSp/10 d.p.i. comparison group; Table S15. Up-regulated GO list of NcSp/20 d.p.i. comparison group; Table S16. Down-regulated GO list of NcSp/20 d.p.i. comparison group; Table S17. Up-regulated KEGG list of NcSp/5 d.p.i. comparison group; Table S18. N.c spore vs. 5 d.p.i. Down regulated KEGG list; Table S19. N.c spore vs. 10 d.p.i. Up regulated KEGG list; Table S20. N.c spore vs. 10 d.p.i. Down regulated KEGG list; Table S21. N.c spore vs. 20 d.p.i. Up regulated KEGG list; Table S22. N.c spore vs. 20 d.p.i. Down regulated KEGG list; Table S23. Up-regulated KEGG list of core genes; Table S24. Down-regulated KEGG list of core genes.

## References

[1] Keeling P. (2009). Five questions about microsporidia. PLoS Pathog..

[2] Bigliardi E., Sacchi L. (2001). Cell biology and invasion of the microsporidia. Microbes Infect..

[3] Keeling P.J., Fast N.M. (2002). Microsporidia: Biology and evolution of highly reduced intracellular parasites. Annu. Rev. Microbiol..

[4] Corradi N., Keeling P.J. (2009). Microsporidia: A journey through radical taxonomical revisions. Fungal Biol. Rev..

[5] Canning E.U. (1988). Nuclear division and chromosome cycle in microsporidia. Biosystems.

[6] Fries I., Martin R., Meana A., García-Palencia P., Higes M. (2006). Natural infections of Nosema ceranae in European honey bees. J. Apic. Res..

[7] Chen Y., Evans J.D., Zhou L., Boncristiani H., Kimura K., Xiao T., Litkowski A., Pettis J.S. (2009). Asymmetrical coexistence of Nosema ceranae and Nosema apis in honey bees. J. Invertebr. Pathol..

[8] Martín-Hernández R., Bartolomé C., Chejanovsky N., Le Conte Y., Dalmon A., Dussaubat C., García-Palencia P., Meana A., Pinto M.A., Soroker V. (2018). Nosema ceranae in Apis mellifera: A 12 years postdetection perspective. Environ. Microbiol..

[9] Huang W.-F., Solter L.F. (2013). Comparative development and tissue tropism of Nosema apis and Nosema ceranae. J. Invertebr. Pathol..

[10] Antúnez K., Martín-Hernández R., Prieto L., Meana A., Zunino P., Higes M. (2009). Immune suppression in the honey bee (*Apis mellifera*) following infection by *Nosema ceranae* (Microsporidia). Environ. Microbiol..

[11] Chen Y.-W., Chung W.-P., Wang C.-H., Solter L.F., Huang W.-F. (2012). Nosema ceranae infection intensity highly correlates with temperature. J. Invertebr. Pathol..

[12] Higes M., Martín R., Meana A. (2006). Nosema ceranae, a new microsporidian parasite in honeybees in Europe. J. Invertebr. Pathol..

[13] Williams G.R., Shutler D., Burgher-MacLellan K.L., Rogers R.E. (2014). Infra-population and-community dynamics of the parasites Nosema apis and Nosema ceranae, and consequences for honey bee (*Apis mellifera*) hosts. PLoS ONE.

[14] Fries I. (2010). Nosema ceranae in European honey bees (*Apis mellifera*). J. Invertebr. Pathol..

[15] Perry C.J., Søvik E., Myerscough M.R., Barron A.B. (2015). Rapid behavioral maturation accelerates failure of stressed honey bee colonies. Proc. Natl. Acad. Sci. USA.

[16] Blažytė-Čereškienė L., Arbačiauskienė V.S., Radžiutė S., Nedveckytė I., Būda V. (2016). Honey bee infection caused by *Nosema* spp. in Lithuania. J. Apic. Sci..

[17] Ilyasov R., Gaifullina L., Saltykova E., Poskryakov A., Nikolaenko A. (2013). Defensins in the honeybee antiinfectious protection. J. Evol. Biochem. Physiol..

[18] Higes M., García-Palencia P., Martín-Hernández R., Meana A. (2007). Experimental infection of *Apis mellifera* honeybees with *Nosema ceranae* (Microsporidia). J. Invertebr. Pathol..

[19] Martín-Hernández R., Botías C., Barrios L., Martínez-Salvador A., Meana A., Mayack C., Higes M. (2011). Comparison of the energetic stress associated with experimental *Nosema ceranae* and Nosema apis infection of honeybees (*Apis mellifera*). Parasitol. Res..

[20] Mayack C., Naug D. (2009). Energetic stress in the honeybee *Apis mellifera* from Nosema ceranae infection. J. Invertebr. Pathol..

[21] Alaux C., Brunet J.L., Dussaubat C., Mondet F., Tchamitchan S., Cousin M., Brillard J., Baldy A., Belzunces L.P., Le Conte Y. (2010). Interactions between Nosema microspores and a neonicotinoid weaken honeybees (*Apis mellifera*). Environ. Microbiol..

[22] Mayack C., Naug D. (2013). Individual energetic state can prevail over social regulation of foraging in honeybees. Behav. Ecol. Sociobiol..

[23] Goblirsch M. (2018). Nosema ceranae disease of the honey bee (*Apis mellifera*). Apidologie.

[24] Jabal-Uriel C., Alba C., Higes M., Rodríguez J.M., Martín-Hernández R. (2022). Effect of Nosema ceranae infection and season on the gut bacteriome composition of the European honeybee (*Apis mellifera*). Sci. Rep..

[25] Paris L., Peghaire E., Mone A., Diogon M., Debroas D., Delbac F., El Alaoui H. (2020). Honeybee gut microbiota dysbiosis in pesticide/parasite co-exposures is mainly induced by *Nosema ceranae*. J. Invertebr. Pathol..

[26] Zhang Y., Lu X., Huang S., Zhang L., Su S., Huang W.-F. (2019). Nosema ceranae infection enhances *Bifidobacterium* spp. abundances in the honey bee hindgut. Apidologie.

[27] Rubanov A., Russell K.A., Rothman J.A., Nieh J.C., McFrederick Q.S. (2019). Intensity of *Nosema ceranae* infection is associated with specific honey bee gut bacteria and weakly associated with gut microbiome structure. Sci. Rep..

[28] Chaimanee V., Chantawannakul P., Chen Y., Evans J.D., Pettis J.S. (2012). Differential expression of immune genes of adult honey bee (*Apis mellifera*) after inoculated by Nosema ceranae. J. Insect Physiol..

[29] Aufauvre J., Misme-Aucouturier B., Viguès B., Texier C., Delbac F., Blot N. (2014). Transcriptome analyses of the honeybee response to Nosema ceranae and insecticides. PLoS ONE.

[30] Badaoui B., Fougeroux A., Petit F., Anselmo A., Gorni C., Cucurachi M., Cersini A., Granato A., Cardeti G., Formato G. (2017). RNA-sequence analysis of gene expression from honeybees (*Apis mellifera*) infected with Nosema ceranae. PLoS ONE.

[31] Huang Q., Wu Z.H., Li W.F., Guo R., Xu J.S., Dang X.Q., Ma Z.G., Chen Y.P., Evans J.D. (2021). Genome and evolutionary analysis of *Nosema ceranae*: A microsporidian parasite of honey bees. Front. Microbiol..

[32] Cornman R.S., Chen Y.P., Schatz M.C., Street C., Zhao Y., Desany B., Egholm M., Hutchison S., Pettis J.S., Lipkin W.I. (2009). Genomic analyses of the microsporidian *Nosema ceranae*, an emergent pathogen of honey bees. PLoS Pathog..

[33] Chang Z.T., Ko C.Y., Yen M.R., Chen Y.W., Nai Y.S. (2020). Screening of Differentially Expressed Microsporidia Genes from *Nosema ceranae* Infected Honey Bees by Suppression Subtractive Hybridization. Insects.

[34] Huang W.-F., Jiang J.-H., Chen Y.-W., Wang C.-H. (2007). A Nosema ceranae isolate from the honeybee *Apis mellifera*. Apidologie.

[35] Huang W.-F., Solter L.F., Yau P.M., Imai B.S. (2013). *Nosema ceranae* escapes fumagillin control in honey bees. PLoS Pathog..

[36] Fries I., Chauzat M.P., Chen Y.P., Doublet V., Genersch E., Gisder S., Higes M., McMahon D.P., Martín-Hernández R., Natsopoulou M. (2013). Standard methods for Nosema research. J. Apic. Res..

[37] Bolger A.M., Lohse M., Usadel B. (2014). Trimmomatic: A flexible trimmer for Illumina sequence data. Bioinformatics.

[38] Schmieder R., Edwards R. (2011). Quality control and preprocessing of metagenomic datasets. Bioinformatics.

[39] Kolde R., Kolde M.R. (2015). Package ‘pheatmap’. R Package.

[40] Oliveros J. (2018). An interactive tool for comparing lists with Venn’s diagrams (2007–2015). https://bioinfogp.cnb.csic.es/tools/venny/index.html.

[41] Moriya Y., Itoh M., Okuda S., Yoshizawa A.C., Kanehisa M. (2007). KAAS: An automatic genome annotation and pathway reconstruction server. Nucleic Acids Res..

[42] Livak K.J., Schmittgen T.D. (2001). Analysis of relative gene expression data using real-time quantitative PCR and the 2^−ΔΔCT^ method. Methods.

[43] R Core Team (2020). R: A Language and Environment for Statistical Computing.

[44] Wickham H., Navarro D., Pedersen T. (2016). ggplot2: Elegant Graphics for Data Analysis.

[45] Kassambara A., Kassambara M.A. (2020). Package ‘ggpubr’. https://rpkgs.datanovia.com/ggpubr/.

[46] Kassambara A., Kosinski M., Biecek P., Fabian S. (2017). Package ‘Survminer’. Drawing Survival Curves Using ‘ggplot2’, R Package version 03.1. http://www.sthda.com/english/rpkgs/survminer/.

[47] Moore D.F. (2016). Applied Survival Analysis Using R..

[48] Spss I. (2011). IBM SPSS Statistics for Windows, version 20.0.

[49] Fontbonne R., Garnery L., Vidau C., Aufauvre J., Texier C., Tchamitchian S., El Alaoui H., Brunet J.L., Delbac F., Biron D.G. (2013). Comparative susceptibility of three Western honeybee taxa to the microsporidian parasite Nosema ceranae. Infect. Genet. Evol..

[50] Paris L., El Alaoui H., Delbac F., Diogon M. (2018). Effects of the gut parasite *Nosema ceranae* on honey bee physiology and behavior. Curr. Opin. Insect Sci..

[51] Dussaubat C., Brunet J.-L., Higes M., Colbourne J.K., Lopez J., Choi J.-H., Martin-Hernandez R., Botias C., Cousin M., McDonnell C. (2012). Gut pathology and responses to the microsporidium *Nosema ceranae* in the honey bee *Apis mellifera*. PLoS ONE.

[52] Goblirsch M., Huang Z.Y., Spivak M. (2013). Physiological and behavioral changes in honey bees (*Apis mellifera*) induced by *Nosema ceranae* infection. PLoS ONE.

[53] Fan Y., Wang J., Yu K., Zhang W., Cai Z., Sun M., Hu Y., Zhao X., Xiong C., Niu Q. (2022). Comparative Transcriptome Investigation of *Nosema ceranae* Infecting Eastern Honey Bee Workers. Insects.

[54] Heinz E., Hacker C., Dean P., Mifsud J., Goldberg A.V., Williams T.A., Nakjang S., Gregory A., Hirt R.P., Lucocq J.M. (2014). Plasma membrane-located purine nucleotide transport proteins are key components for host exploitation by microsporidian intracellular parasites. PLoS Pathog..

[55] Vidau C., Panek J., Texier C., Biron D.G., Belzunces L.P., Le Gall M., Broussard C., Delbac F., El Alaoui H. (2014). Differential proteomic analysis of midguts from *Nosema ceranae*-infected honeybees reveals manipulation of key host functions. J. Invertebr. Pathol..

[56] Vávra J., Ronny Larsson J. (1999). Structure of the microsporidia. The Microsporidia and Microsporidiosis.

[57] He Q., Vossbrinck C.R., Yang Q., Meng X.-Z., Luo J., Pan G.-Q., Zhou Z.-Y., Li T. (2019). Evolutionary and functional studies on microsporidian ATP-binding cassettes: Insights into the adaptation of microsporidia to obligated intracellular parasitism. Infect. Genet. Evol..

[58] Nelson N. (1999). Metal ion transporters and homeostasis. EMBO J..

[59] Freisinger E., Sigel R.K. (2007). From nucleotides to ribozymes—A comparison of their metal ion binding properties. Coord. Chem. Rev..

[60] Sigel R., Sigel H. (2013). Metal ion interactions with nucleic acids and their constituents. Compr. Inorg. Chem. II.

[61] Schweins T., Wittinghofer A. (1994). GTP-binding proteins: Structures, interactions and relationships. Curr. Biol..

[62] Hochstrasser M. (1996). Ubiquitin-dependent protein degradation. Annu. Rev. Genet..

[63] Kim I.H., Kim D.J., Gwak W.S., Woo S.D. (2020). Increased survival of the honey bee Apis mellifera infected with the microsporidian *Nosema ceranae* by effective gene silencing. Arch. Insect Biochem. Physiol..

[64] Yang D., Pan L., Chen Z., Du H., Luo B., Luo J., Pan G. (2018). The roles of microsporidia spore wall proteins in the spore wall formation and polar tube anchorage to spore wall during development and infection processes. Exp. Parasitol..

[65] Wu Z., Li Y., Pan G., Tan X., Hu J., Zhou Z., Xiang Z. (2008). Proteomic analysis of spore wall proteins and identification of two spore wall proteins from *Nosema bombycis* (Microsporidia). Proteomics.

[66] Wu Z., Li Y., Pan G., Zhou Z., Xiang Z. (2009). SWP25, a novel protein associated with the *Nosema bombycis* endospore 1. J. Eukaryot. Microbiol..

[67] Ni W., Bao J., Mo B., Liu L., Li T., Pan G., Chen J., Zhou Z. (2020). Hemocytin facilitates host immune responses against *Nosema bombycis*. Dev. Comp. Immunol..

[68] Waterhouse A., Bertoni M., Bienert S., Studer G., Tauriello G., Gumienny R., Heer F.T., de Beer T.A.P., Rempfer C., Bordoli L. (2018). SWISS-MODEL: Homology modelling of protein structures and complexes. Nucleic Acids Res..

[69] Spagnolo L., Rivera-Calzada A., Pearl L.H., Llorca O. (2006). Three-dimensional structure of the human DNA-PKcs/Ku70/Ku80 complex assembled on DNA and its implications for DNA DSB repair. Mol. Cell.

[70] Mazet F., Yu J.-K., Liberles D.A., Holland L.Z., Shimeld S.M. (2003). Phylogenetic relationships of the Fox (Forkhead) gene family in the Bilateria. Gene.

[71] Partridge L., Brüning J. (2008). Forkhead transcription factors and ageing. Oncogene.

[72] Ohi M.D., Link A.J., Ren L., Jennings J.L., McDonald W.H., Gould K.L. (2002). Proteomics analysis reveals stable multiprotein complexes in both fission and budding yeasts containing Myb-related Cdc5p/Cef1p, novel pre-mRNA splicing factors, and snRNAs. Mol. Cell. Biol..

[73] Kim D.-U., Maeng S., Lee H., Nam M., Lee S.-J., Hoe K.-L. (2016). The effect of the cwf14 gene of fission yeast on cell wall integrity is associated with rho1. J. Microbiol..

[74] Meier U.T. (2005). The many facets of H/ACA ribonucleoproteins. Chromosoma.

[75] Mayes A.E., Verdone L., Legrain P., Beggs J.D. (1999). Characterization of Sm-like proteins in yeast and their association with U6 snRNA. EMBO J..

[76] Green M.R. (1986). Pre-mRNA splicing. Annu. Rev. Genet..

