# Supplementary material for: Transcriptome of Nosema ceranae and Upregulated Microsporidia Genes during Its Infection of Western Honey Bee (Apis mellifera)
Source: Insects. 2022 Aug 9;13(8):716. doi: 10.3390/insects13080716 (PMC9409478; doi:10.3390/insects13080716)
Supplement: Supplementary file 1 [file insects-13-00716-s001.zip › 20220610_Supplementary_Figures.pdf]

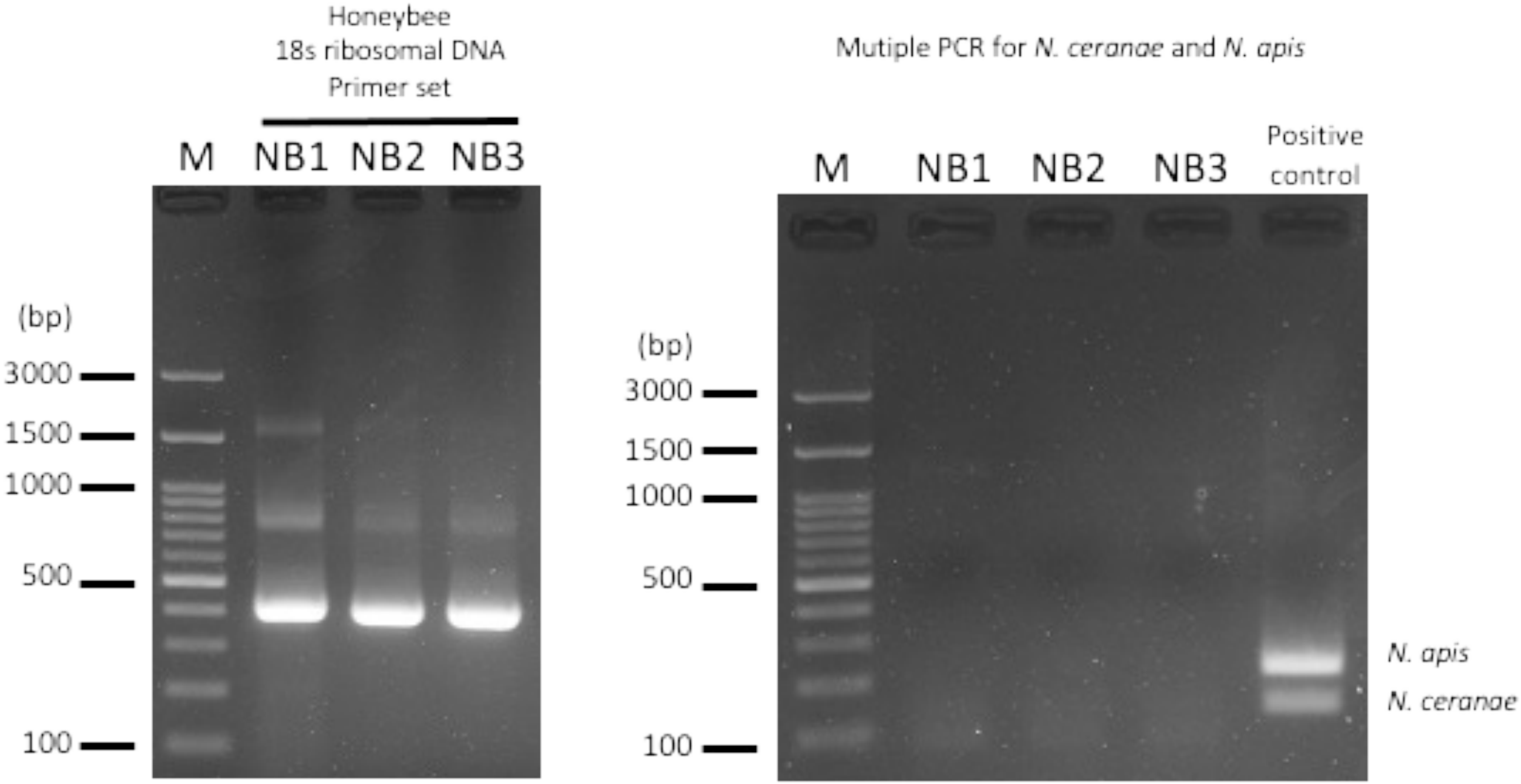

Supplementary Figure S1. The detection of *Nosema* spp. of newly emerged adult honey bees by multiplex PCR before experiment. The information of primer sets were listed in Supplementary Table 1.

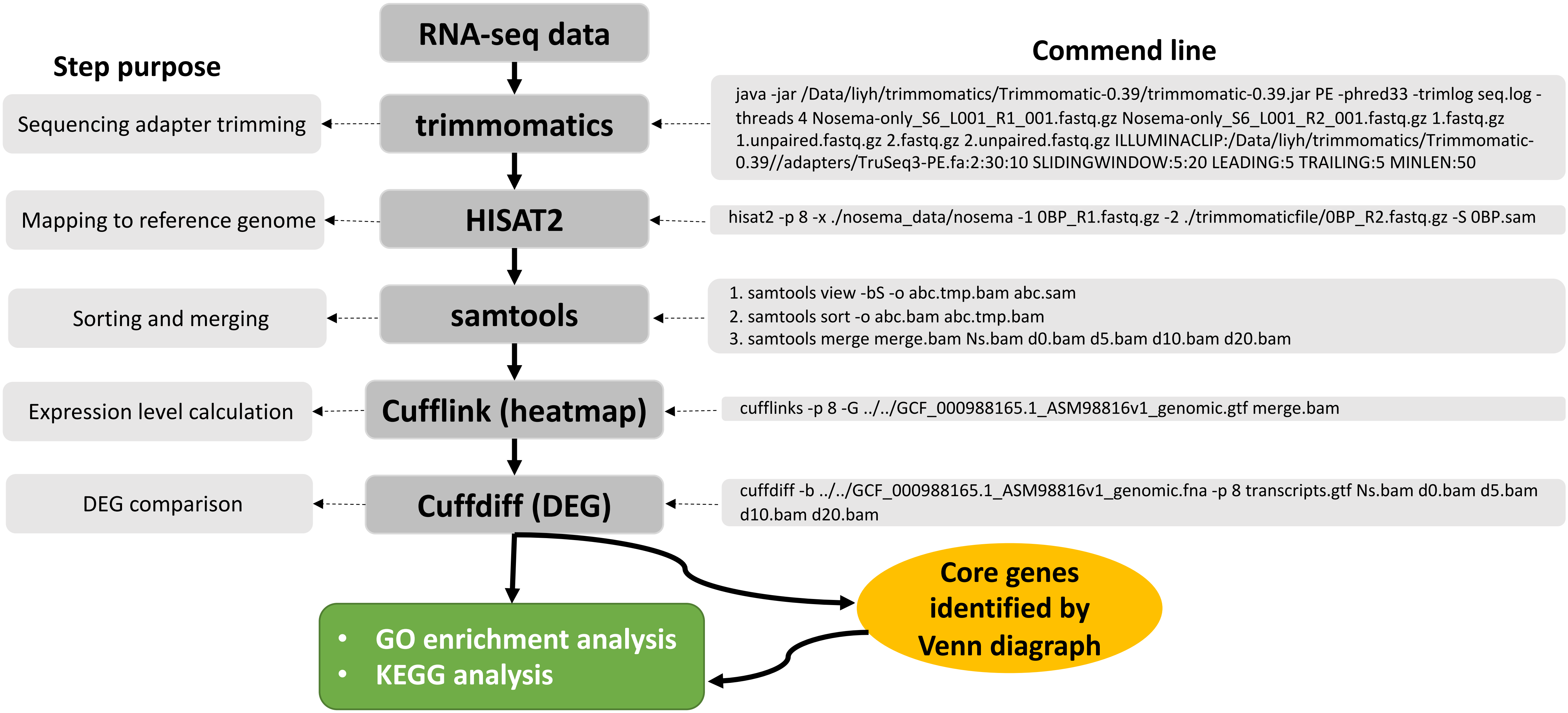

Supplementary Figure S2. The pipeline of transcriptome analysis. The commend lines and analysis purposes are noted pairwise the item. DEG= differential expressed gene; FPKM= fragment per kilobase per million; GO= gene ontology; KEGG= Kyoto Encyclopedia of Genes and Genomes.
